# 

**Published:** 1887-07-09

**Authors:** 


### THE HOSPITAL

An Institution, Family, and Parochial Journal of Hospitals, Asylums, & all Agencies for the Care of the Sick, Criticism & News.

### CONTENTS.

**Begging Parsons** - - - - - - - 241

**Words of Consolation.**—XL. The Work of the Holy Ghost - - - - - - - 242

**Norfolk and Norwich Hospital.**—A Correction - 242

**Counterparts.** By a Student of Eastern Philosophies.—Chap. V. How it all Ended - - - 243

**Children's Descriptions of Jubilee Celebrations** - - - - - - - 244

**A Noble Profession: Nursing the Sick.**—The Nurse and the Sick-room. By Miss Mollett (*concluded*) - - - - - - - 245

**The National Pension Fund** - - - - 247

**Notes and News.**—Miscellaneous Hospital Intelligence.—Vacancies.—The Duke of Cambridge and the Chelsea Royal Hospital.—Prize Distribution at Charing Cross Hospital.—Hospital Resources.—Hospital Saturday Fund State Procession and Demonstration.—The Prince of Wales and the Sunderland Infirmary.—"The Repeated Jubilee Service" at Westminster Abbey.—The North-Eastern Hospital for Children. — Provident Dispensaries and the Hospitals, etc., etc. - - - 248

**Annotations.**—Wooden Hospitals.—Convalescent Aid.—A Krupp Gun Wanted - - - - - 251

**Doctors in Fiction** - - - - - 252

**Nerves: their Uses and Troubles** - - - 253

**Old London Houses** - - - - - 254

**The Editor's Letter-Box.**—The Chadwick Home Hospital, Liverpool.—The Pension Fund for Nurses.—More Knowledge and Thought Wanted - - - 254

**The Children's Corner.**—Puzzles, Answers, etc. - 255

**Amusements with Profit** - - - - 255

**In- and Out-Patients' Hospital Diary** - - 256

